# Physical activity and depressive symptoms among college students: positive mental health as a mediator

**DOI:** 10.3389/fpsyg.2026.1704433

**Published:** 2026-01-22

**Authors:** Yizhou Chen, Jie Zhang

**Affiliations:** 1 Department of Physical Education, Quzhou University, Quzhou, Zhejiang, China; 2 College of Physical Education, Anhui Normal University, Wuhu, Anhui, China

**Keywords:** college students, depressive symptoms, path analysis, physical activity, positive mental health

## Abstract

**Introduction:**

Numerous studies confirm that physical activity alleviates depressive symptoms in college students, but the underlying mechanisms remain unclear. This study aimed to explore whether positive mental health mediates the relationship between physical activity and depressive symptoms.

**Methods:**

A cross-sectional online survey was conducted among 3,140 college students, who completed self-report questionnaires on physical activity, positive mental health, and depressive symptoms. Data were analyzed using SPSS 29.0 and AMOS 29.0 with structural equation modeling.

**Results:**

Results showed 69.39% of participants had low physical activity, and 19.24% reported clinically significant depressive symptoms. The findings showed significant correlations among the three variables, with positive mental health strongly associated with reduced depressive symptoms. Bootstrap mediation analysis (5,000 iterations) confirmed a mediating role of positive mental health.

**Discussion:**

This study advances theoretical understanding by identifying positive mental health as one possible pathway linking physical activity to better mental health outcomes. Due to the effect is small, the results should be interpreted cautiously. The findings provide conceptual support for integrating positive mental health promotion into physical activity-based interventions targeting college students’ depressive symptoms, with no causal inferences implied due to the cross-sectional design.

## Introduction

Depressive symptoms, a prevalent manifestation of mental health conditions that encompass a cluster of negative emotional, cognitive, and behavioral experiences persisting for a specific duration, mainly feature lasting emotional distress and a notable reduction in pleasure from activities that were previously enjoyable (Ding et al., 2024; Xiang et al., 2025). Depressive symptoms constitute a pressing global public health challenge and stand as the most prevalent mental health disorder among college students (Li et al., 2022; Vieira et al., 2021). College students experiencing depressive symptoms face not only dual impairments to their physical and mental health—such as persistent somatic discomfort like poor sleep quality and difficulty falling asleep (Dağ and Kutlu, 2017), gastrointestinal dysfunction (Wouters and Boeckxstaens, 2015), alongside cognitive and emotional issues including feelings of worthlessness or excessive guilt (Akkın Gürbüz et al., 2016), and high suicidal ideation (Omary, 2021)—but also practical difficulties including poor quality of life (Alyahya et al., 2025), low academic achievements (Quinn et al., 2023), and poor social feelings (Jelsma et al., 2024).

Given the profound impact of depressive symptoms on college students’ academic performance, quality of life and well-being, identifying factors that can mitigate or alleviate these symptoms is crucial. Among numerous potential factors, physical activity has emerged as a promising candidate due to its well-documented mental health benefits (Brown and Kwan, 2021; Rashidi et al., 2024), with its positive role in preventing and mitigating depressive symptoms gaining widespread consensus within the global research community (Ansari et al., 2025; Kawai and Yamagata, 2024; Kim and Chung, 2021; Zhang and Tian, 2022). However, exactly through what psychological mechanisms physical activity exerts its positive effects remains a key issue that current research is striving to reveal. Traditional explanations have mostly focused on the direct relief of negative emotions and stress through physical activity (Ghrouz et al., 2019; Yang et al., 2023), while relatively neglecting the potential path of combating depression by cultivating positive psychological qualities. In recent years, the introduction of the perspective of positive psychology has expanded our understanding of mental health (Subotic-Kerry et al., 2023). Positive mental health not only means the absence of mental disorders, but also emphasizes the possession of positive emotional experiences, psychological resilience, and a sense of control over life, among other positive resources (Phua and Chew, 2025). Research has shown that physical activity can effectively enhance an individual’s positive mental health (Lenze et al., 2025); moreover, robust positive mental health itself has been proven to be a powerful protective factor against depressive symptoms (Keyes, 2006). A study on female academic students indicated that physical activity habits can improve their self-assessment levels of physical and mental health, thereby reducing psychological distress (Levante et al., 2024). Another study indicated that individuals with higher levels of physical activity have lower psychological vulnerability and stronger psychological adaptability, thus exhibiting lower levels of mental health problems (such as depression) (Wang Z. et al., 2025). This suggests that the benefits of physical activity for depressive symptoms may be achieved, in part, by nourishing and enhancing the individual’s internal resource of positive mental health. To empirically test this hypothesized mediating pathway, the present study employs path analysis to examine whether and how physical activity reduces depressive symptoms through the enhancement of positive mental health among university students. By elucidating these interrelationships, this research aims to advance the current understanding of mental health dynamics in the college population. Next, this paper will first systematically review the theoretical foundations and empirical studies related to physical activity, positive mental health, and depressive symptoms, then clarify the theoretical mechanisms of the mediating role of positive mental health, and finally put forward the research hypotheses to be tested in this study.

## Theoretical background and hypotheses

### Theoretical framework

The relationship between physical activity, positive mental health, and depressive symptoms among college students is anchored in Self-Determination Theory (SDT), a macro-theory of motivation and personality that identifies autonomy, competence, and relatedness as three innate psychological needs critical to high-quality motivation and psychological well-being (Ryan and Deci, 2000). For this study, SDT provides a targeted mechanism linking physical activity to mental health outcomes by emphasizing psychological (rather than purely physiological) pathways, with three core connections to the research variables: First, physical activity functions as a context for basic psychological need satisfaction among college students. Its mental health impact is contingent on whether it fulfills (rather than thwarts) these needs: autonomy is satisfied when students engage in physical activity volitionally (Teixeira et al., 2012); competence stems from mastering movement challenges and perceiving progress; and relatedness is fostered through social connection in team sports, group fitness, or casual exercise settings (Ntoumanis et al., 2021). Second, SDT defines the mediating construct of positive mental health in this study as a state of eudaimonic well-being and vitality—outcomes directly tied to need satisfaction (Ryan and Deci, 2001). Vitality, a key indicator for active populations, refers to a feeling of aliveness and energy that is heightened by need-supportive physical activity (Ryan and Frederick, 1997), distinguishing it from vague positive affect and aligning it with measurable mental health outcomes. Third, SDT explains the link to reduced depressive symptoms via the concept of need frustration. Persistent thwarting of autonomy (perceived control), competence (feelings of inadequacy), and relatedness (social isolation) is a primary antecedent of depressive symptomatology (Vansteenkiste and Ryan, 2013). Thus, the positive mental health cultivated through need-satisfying physical activity acts as a buffer against depressive risk. In sum, the study’s conceptual model, derived from SDT, posits the following chain: physical activity facilitates the satisfaction of autonomy, competence, and relatedness needs among college students; this need satisfaction enhances positive mental health (eudaimonia and vitality); and this elevated well-being ultimately mitigates depressive symptom risk.

### Physical activity and depressive symptoms

Physical activity is defined by the World Health Organization (2024) as any skeletal muscle-powered bodily movement involving detectable energy expenditure, including both planned exercise and incidental physical activity. Beyond well-documented benefits for physical health, insufficient physical activity has been identified as a modifiable contributor to diminished psychological wellness and increased risk of mental health issues globally (Andrade, 2024; Borodulin and Anderssen, 2023). From the perspective of Self-Determination Theory (SDT) (Ryan and Deci, 2000), a prominent framework for understanding human motivation and well-being, physical activity engagement inherently interacts with three basic psychological needs—autonomy, competence, and relatedness—that are critical for mental health. When individuals engage in physical activity that fosters a sense of choice (autonomy), mastery of skills or challenges (competence), and social connection (relatedness), their intrinsic motivation is enhanced, which in turn promotes psychological thriving and mitigates negative emotional states. Extant empirical evidence aligns with this theoretical premise: systematic reviews confirm that moderate physical activity reduces depressive symptoms among children and adolescents (Eime et al., 2013), while prospective studies link sustained physical activity to lower depressive symptom load and sedentary behavior to increased risk (Kandola et al., 2020). For college students specifically, light-to-moderate aerobic exercise has been shown to alleviate depressive symptoms when sustained (Herbert, 2022), a effect likely mediated by the satisfaction of basic psychological needs. Integrating SDT’s theoretical insights with empirical findings, this study proposes that physical activity’s association with depressive symptoms is rooted in its potential to fulfill core psychological needs. Thus, we hypothesize:

*Hypothesis 1*: Physical activity is negatively correlated with depressive symptoms among college students.

### Positive mental health as a mediator

Within positive psychology frameworks, positive mental health refers to a comprehensive psychological state that encompasses continuous positive emotional experiences (such as well-being), key psychological resource traits (such as self-esteem, sustained hedonic, and self-control), and contextually adaptive stressor-response patterns. In this state, individuals can fully recognize their own potential, effectively cope with life stress, maintain high-functioning social contributions, and achieve collaborative well-being for both the individual and the collective (World Health Organization, 2005). Numerous studies demonstrated that physical activity is positively correlated with positive mental health (Brown and Kwan, 2021; Chow and Choi, 2019; Domingues, 2018; Surprenant et al., 2025). A study of 7,539 participants from the Irish population found that participants meeting recommended physical activity levels demonstrated, on average, threefold higher positive mental health scores compared to inactive counterparts (Bowe et al., 2019). A prospective Canadian adolescent study revealed that daily moderate-to-vigorous activity indirectly enhances positive mental health, mediated by greater outdoor time (Bélanger et al., 2019). Extensive research documents that elevated levels of positive mental health confer multifaceted psychosocial benefits, such as improving interpersonal functioning and quality of life, enhancing well-being and life satisfaction, and even significantly alleviating depressive symptoms and suicidal thoughts (Arslan and Öztürk, 2025; Burns and Fard, 2021; Garg and Kharb, 2024; Kang et al., 2020; Kotera et al., 2022; Sambasivam et al., 2016; Seow et al., 2016; Tafoya and Aldrete-Cortez, 2018). Research evidence demonstrates a significant inverse association between positive mental health and depressive symptoms (Deis et al., 2025). An investigation involving 1,234 youth examining subjective well-being revealed dose–response gradients across the mental health continuum: as individuals’ mental health progressed from languishing to flourishing states, psychosocial functioning markers (e.g., self-determination capacity, relational closeness) increased significantly, with parallel reductions in depressive symptomatology (Keyes, 2006). Building upon demonstrated connections among physical activity, positive mental health, and depressive symptoms, the second hypothesis appears theoretically justified:

*Hypothesis 2*: Positive mental health mediates the relationship between physical activity and depressive symptoms.

**Figure 1:**
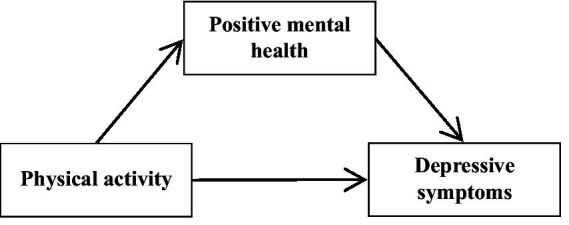
The proposed conceptual framework.

This study examined the relationship between physical activity and depressive symptoms, with a focus on the mediating role of positive mental health. The proposed conceptual framework is presented in Figure 1.

## Methods

### Participants and procedures

This cross-sectional study employed a convenience sampling method to collect data from Chinese college students via the Wenjuanxing online survey platform^1^ between May and June 2025. The data collection procedure was implemented as follows: First, collaboration was established with faculty members from multiple universities in China, who were provided with the electronic questionnaire link along with clear inclusion criteria for participants. Second, the collaborating faculty distributed the questionnaire link to college students through their administrative class groups or course-related WeChat groups, QQ groups, or DingTalk groups (the three most popular interaction platforms among Chinese college students), inviting voluntary participation. Third, an informed consent statement was presented on the first page of the questionnaire, detailing the study purpose, data usage, and privacy protection measures. Participants could only access the formal questionnaire by clicking “Agree to Participate,” which was deemed equivalent to signing the informed consent form. Data included: (a) demographics (gender, age, etc.), and (b) standardized measures of physical activity volume, positive mental health, and depressive symptoms. Inclusion criteria: (i) full-time students aged 18 and above enrolled in Chinese mainland colleges; (ii) no severe physical injuries (e.g., fractures, ankle sprains) in the past month; (iii) provided complete responses on all standardized measures without missing values. Exclusion criteria: (i) self-reported diagnosis of major depressive disorder or other psychiatric conditions by healthcare professionals prior to participation; (ii) currently matriculated in a graduate degree program (master’s or PhD); (iii) degree-seeking international students attending higher education institutions in mainland China. The initial data collection yielded 3,215 valid responses from the target population prior to quality screening. After applying predefined inclusion/exclusion criteria, we removed 75 ineligible responses (2.33%), including: (a) abnormally short (<4 min) or long (>12 min) completion times, (b) straight-lined responses, and (c) other protocol violations. The final analytic sample comprised 3,140 participants from 22 provinces in China, representing a 97.67% response rate. This sample size substantially exceeds minimum requirements, aligning with prevailing methodological norms (Sousa and Rojjanasrirat, 2011). Participants were aged 18–27 years (mean ± SD: 20.02 ± 1.28). Table 1 summarizes the coding structure and distributional characteristics of all demographic variables.

**Table 1:** Coding structure and distributional characteristics of all demographic variables (*n* = 3,140).

Variable	Coding structure	Categorical variable	*n* (%)
Gender	1	Male	1,478 (47.07)
	2	Female	1,662 (52.93)
Grade	1	Freshman	1,124 (35.80)
	2	Sophomore	1,180 (37.58)
	3	Junior	709 (22.58)
	4	Senior	127 (4.04)
Major	1	Liberal arts	1,539 (49.01)
	2	Science	1,601 (50.99)
Educational level^a^	1	Junior college students	761 (24.24)
	2	Undergraduate students	2,379 (75.76)

^a^Educational level: “junior college students” refers to 3 year vocational programs in China, while “undergraduate students” denotes 4-year bachelor’s degree programs.

### Ethics statement

The research protocol received institutional ethical validation (Ethics Number: AHNU-ET 2025111, May 28, 2025), confirming alignment with international biomedical research standards for human participant protection. Before data collection, each participant received and voluntarily signed a clear electronic informed consent form, which detailed the research purpose, methods, potential risks/benefits, data confidentiality measures, with guaranteed unrestricted discontinuation privileges. All the data were anonymized, encrypted, and stored on a password-protected server. In the event of participant withdrawal, their data would be promptly sealed or deleted to ensure data confidentiality. The participants did not receive any compensation for participating in this study.

### Measures

#### Physical activity

This current study measured physical activity used the Physical Activity Rank Scale-3 (PARS-3), which was developed by Hashimoto (1990) and revised by Liang (1994). The PARS-3 comprises three sequential items: intensity (physical activity intensity in the past month), duration (length of an activity at the specified intensity), and frequency (monthly occurrence of such activities). The scoring range for the “intensity” and “frequency” items is 1 to 5 points, while the scoring range for the “duration” item is 0 to 4 points. The product of the scores for these three items represents the total score of the physical activity, with a total score range of 0 to 100. The criteria for determining exercise volume are as follows: a score of ≤19 indicates low exercise volume, 20–42 indicates moderate exercise volume, and ≥43 indicates high exercise volume (Liang, 1994). In the present study, the PARS-3 demonstrated a Cronbach’s alpha coefficient of α = 0.634.

While this value falls below the conventional threshold of 0.7 for confirmatory research (Nunnally and Bernstein, 1994), it aligns with multiple empirical studies that have validated the scale for use in Chinese community samples (0.7 > α > 0.6; e.g., Wang W. et al., 2025; Yang et al., 2025). Notably, the marginal internal consistency can be partly attributed to the scale’s three-item, multiplicative scoring structure. Unlike additive scales, multiplicative scoring reduces inter-item linearity, which typically lowers Cronbach’s alpha values (Streiner et al., 2003). Furthermore, a threshold of α ≥ 0.6 is widely accepted in physical activity epidemiology for exploratory research aimed at identifying preliminary associations—the core objective of this study—as it balances measurement feasibility with preliminary data utility, especially for understudied populations such as college students (Wang W. et al., 2025; Yang et al., 2025). However, it is important to acknowledge the associated limitation. This level of internal consistency could potentially attenuate observed correlations and mediation effects, possibly leading to an underestimation of the true relationships under investigation. Alternatively, the measurement error introduced might act as a source of noise, complicating the interpretation of smaller effect sizes. This limitation is considered when interpreting the study’s findings.

#### Positive mental health

The current investigation assessed participants’ positive mental health using the validated Positive Mental Health Scale (Lukat et al., 2016). This unidimensional 9-item scale (e.g., “I enjoy my life”) used a 4-point Likert scale (1 = “*not sure*” to 4 = “*sure*”). Total scores represented overall positive mental health, with higher scores indicating greater positive mental health. The scale demonstrated excellent reliability in this study (α = 0.95), consistent with prior validations in Chinese populations (Liu et al., 2025; Margraf et al., 2023).

#### Depressive symptoms

The Patient Health Questionnaire-9 (PHQ-9), developed by Kroenke’s research team (Kroenke et al., 2001), serves as a rigorously validated self-report measure originally designed for depression screening among clinical patients in hospital settings. Renowned for its brevity, efficiency, and robust psychometric properties, it has gained widespread recognition across the international medical, psychological, and public health communities (Alreshidi, 2024; Begashaw and Andualem, 2024; Lee and Lee, 2024; Newman, 2022; Pellas and Damberg, 2021; Storz, 2025), and its application has extended to non-clinical populations, including the general public and students (Kliem et al., 2024; Zhang et al., 2013). Comprising 9 items (e.g., “poor appetite or overeating”), the PHQ-9 asks respondents to rate their experiences over the past 2 weeks using a 4-point Likert scale: 0 (“Not at all”), 1 (“Several days”), 2 (“More than half the days”), and 3 (“Nearly every day”). The instrument yields a total score ranging from 0 (asymptomatic) to 27 (maximum severity), where elevated scores indicate worsening depressive symptomatology. A threshold score of ≥11 demonstrates optimal sensitivity for identifying probable clinical depression (Zhang et al., 2013). Extensive research supports the Chinese PHQ-9’s cultural adaptation (Gao and Liu, 2024; Yan and Wang, 2025; Zhang et al., 2013). The current study found the measure to be highly consistent (α = 0.935).

### Statistical analysis

All statistical analyses were conducted using IBM SPSS Statistics 29 and AMOS 29. Data from all 3,140 participants were complete and included in the analysis. Common method bias was first assessed prior to examining the latent structural equation model. Subsequently, descriptive statistics (including means, standard deviations, skewness, and kurtosis) were computed to characterize the distribution of core variables, and bivariate correlation analyses were conducted to explore preliminary associations among variables. A rigorous normality evaluation was implemented for all continuous variables (i.e., physical activity volume, positive mental health scores, and depressive symptoms scores) through a multi-dimensional approach: Kolmogorov–Smirnov (K-S) test, distributional indices (skewness and kurtosis), and visual inspections (histograms with normal curve overlays and Q-Q plots). The results showed that the K-S test (a method well-suited for large samples, *n* ≥ 50) yielded statistically significant results for all continuous variables (all *p* < 0.001), indicating deviations from strict normality. Further analysis of distributional indices revealed variable-specific non-normal characteristics: (1) Physical activity volume exhibited significant right skewness [skewness = 1.736, standard error (SE) = 0.044] and pronounced leptokurtosis (kurtosis = 2.689, SE = 0.087), reflecting a severe departure from normality—this was attributed to the large proportion of participants with low exercise volume [69.39% scored ≤ 19, per Liang’s (1994) criteria]; (2) Positive mental health scores showed mild left skewness (skewness = −0.279, SE = 0.044) and slight leptokurtosis (kurtosis = 1.091, SE = 0.087); (3) Depressive symptoms scores displayed mild right skewness (skewness = 0.844, SE = 0.044) and modest leptokurtosis (kurtosis = 1.260, SE = 0.087). Notably, an increase in skewed items can lead to more significant biases in large samples (Xiao and Hau, 2023). To address this issue, robust non-parametric approaches were adopted for primary analyses: Spearman’s rank correlation (instead of Pearson correlation) was used to quantify bivariate associations, as it is insensitive to non-normal distributions by focusing on variable ranks (Field, 2024). Mediation analysis was performed using the PROCESS macro (version 4.1) for SPSS, developed by Hayes (2018). This tool was specifically selected for three key reasons: First, it allows for the inclusion of covariates to control for confounding effects; second, its bias-corrected bootstrap procedure is inherently robust to non-normal data, as it estimates effect distributions by resampling the original dataset rather than relying on asymptotic normality assumptions (Hayes, 2018); third, it has become a gold standard for mediation testing in social and behavioral sciences, ensuring methodological consistency with prior literature. The significance of indirect effects was tested using a bias-corrected bootstrap approach with 5,000 resamples. An effect was considered statistically significant if the 95% confidence interval (CI) did not include zero. This criterion minimizes biases in inference caused by non-normal effect distributions and enhances the reliability of mediation results (Preacher and Hayes, 2008). This analysis controlled for age, gender, grade, and educational level, as these covariates have established associations with depression in prior literature (e.g., Abu-Kaf and Khalaf, 2020; Cho et al., 2024; Dou and Feng, 2025; El Ansari and Berg-Beckhoff, 2019; Feng and Dou, 2024).

## Results

### Common method bias and structural equation model fit testing

To assess common method bias, this study performed a single-factor test using principal component analysis. The analysis yielded three components (eigenvalues > 1.0), with the largest explaining 37.37% of variance—below the 40% threshold (Podsakoff et al., 2003), suggesting minimal method bias. Multicollinearity was examined using variance inflation factors (VIF). All VIF values were 1.013, which are substantially below the conservative threshold of 5 (Hair et al., 2011), indicating that multicollinearity is not a significant issue in this model.

A sequence of confirmatory factor analyses (CFA) was performed in Amos 29.0 to examine the distinctiveness of the latent constructs. The analysis compared the fit of the hypothesized three-factor model to more constrained, nested alternatives: a two-factor model (with physical activity and depressive symptoms loading on a single factor) and a one-factor model. Goodness-of-fit was evaluated using established indices, including χ^2^/df, CFI, TLI, GFI, and RMSEA (Ferreira-Valente et al., 2016). The results indicated that the three-factor model provided a significantly better fit to the data than the competing models (Table 2), offering robust evidence for discriminant validity.

**Table 2:** Comparison of fit indices for latent structural equation models.

Model	χ^2^/df	CFI	TLI	GFI	RMSEA
Single-factor model Integrating PA, PMH, and DS	120.692	0.533	0.481	0.420	0.195
Two-factor model Integrating PA and DS	14.616	0.952	0.941	0.924	0.066
Three-factor model PA, PMH, and DS	2.677	0.996	0.993	0.991	0.023

PA, Physical activity; PMH, Positive mental health; DS, Depressive symptoms.

### Preliminary analyses

Among the 3,140 participants, the mean scores for physical activity, positive mental health, and depressive symptoms were 18.50 ± 21.37, 26.42 ± 5.21, and 7.60 ± 5.25, respectively. According to the depression screening criteria (PHQ-9 ≥ 11), 604 students (19.24%) were identified as having depressive symptoms. In terms of activity levels, the majority of participants (69.39%, *n* = 2,179) reported exercise volume, followed by moderate (16.15%, *n* = 507) and high (14.46%, *n* = 454) activity levels.

The Spearman’s correlation matrix among variables are shown in Table 3. Age demonstrated a significant negative association with physical activity (*ρ* = −0.054, *p* < 0.01), but no significant correlations emerged between age and positive mental health (*ρ* = −0.009, *p* > 0.05), or depressive symptoms (*ρ* = −0.011, *p* > 0.05). Gender demonstrated significant negative associations with physical activity (*ρ* = −0.273, *p* < 0.001), positive mental health (*ρ* = −0.071, *p* < 0.001), but no significant correlations with depressive symptoms (*ρ* = −0.025, *p* > 0.05). Grade demonstrated a significant negative association with physical activity (*ρ* = −0.099, *p* < 0.001), whereas no significant correlations with positive mental health (*ρ* = −0.019, *p* > 0.05), or depressive symptoms (*ρ* = 0.002, *p* > 0.05). No significant correlations emerged between major and physical activity (*ρ* = 0.018, *p* > 0.05), positive mental health (*ρ* = 0.023, *p* > 0.05), or depressive symptoms (*ρ* = 0.007, *p* > 0.05). Educational level demonstrated a significant positive association with depressive symptoms (*ρ* = 0.042, *p* < 0.05), whereas no significant associations were observed between educational level and physical activity (*r* = 0.029, *p* > 0.05), or positive mental health (*ρ* = −0.022, *p* > 0.05). Physical activity showed divergent associations—a small but significant positive link to positive mental health (*ρ* = 0.143, *p* < 0.001) contrasted with a weaker negative connection to depressive symptoms (*ρ* = −0.089, *p* < 0.001). Moreover, positive mental health was inversely correlated with depressive symptoms (*ρ* = −0.362, *p* < 0.001).

**Table 3:** Spearman rank-order correlation matrix (*n* = 3,140).

Variables	Age	Gender	Grade	Major	Educational level	Physical activity	Positive mental health	Depressive symptoms
Age	1							
Gender	−0.002	1						
Grade	0.762***	0.062***	1					
Major	−0.062***	−0.358***	−0.024	1				
Educational level	0.193***	0.202***	0.206***	−0.097***	1			
Physical activity	−0.054**	−0.273***	−0.099***	0.018	0.029	1		
Positive mental health	−0.009	−0.071***	−0.019	0.023	−0.022	0.143***	1	
Depressive symptoms	−0.011	−0.025	0.002	0.007	0.042*	−0.089***	−0.362***	1
*M* (SD)	20.02 (1.28)	1.53 (0.50)	1.95 (0.86)	1.51 (0.50)	1.76 (0.43)	18.50 (21.37)	26.42 (5.21)	7.60 (5.25)

M, Mean; SD, Standard Deviation. *p < 0.05, **p < 0.01, and ***p < 0.001.

### Positive mental health as a mediator

**Figure 2:**
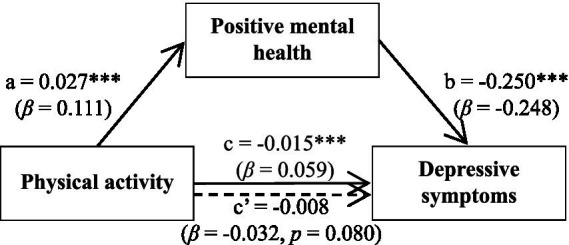
Path coefficients for the mediated relationship of physical activity with depressive symptoms through positive mental health. Path values are unstandardized coefficients with standardized coefficients (β) in parentheses. The c path represents the total effect of physical activity on depressive symptoms, c’ path represents the direct effect, and a and b paths represent the two legs of the indirect effect through positive mental health. The indirect effect (*a × b*) was tested using 5,000 bias-corrected bootstrap samples. All paths control for age, gender, grade, and educational level. **p < 0.01, ***p < 0.001.

A mediation analysis was conducted using Model 4 from the PROCESS macro (Hayes, 2018) in SPSS 29.0, wherein positive mental health was tested as a mediator between physical activity and depressive symptoms. The 95% CI for the indirect effect was based on 5,000 bias-corrected bootstrap samples. In the present study, age, gender, grade, major, and educational level were treated as control variables. The total effect of physical activity on depressive symptoms (the **c path**) was significant, *c* = −0.015, *p* < 0.01, 95% CI [−0.024, −0.006]. When the mediator was included in the model, physical activity showed a positive association with positive mental health (the **a path**), *a* = 0.027, *p* < 0.001, 95% CI [0.018, 0.036]. It is noteworthy that this significant *a* path was identified within a model that explains a limited portion of variance in the mediator (*R*^2^ = 0.014 for positive mental health), underscoring that physical activity is one of many potential contributors to positive mental health. Positive mental health, in turn, was negatively associated with depressive symptoms (the **b path**), *b* = −0.250, *p* < 0.001, 95% CI [−0.284, −0.216]. The direct effect of physical activity on depressive symptoms (the **c’ path**) became non-significant, *c’* = −0.008, *p* = 0.080, 95% CI [−0.017, 0.001]. The indirect effect (*a × b*) through positive mental health was significant, *ab* = −0.007, 95% CI [−0.010, −0.004]. These results indicate that positive mental health mediates the relationship between physical activity and depressive symptoms (see Table 4; Figure 2). Although the unstandardized effect size appears numerically small, it represents a meaningful relationship when considered in context (see Discussion for implications).

**Table 4:** Summary of effects in the mediation model.

Predictors	Positive mental health			Depressive symptoms		
	β	*SE*	*95% CI*	β	*SE*	95% CI
Age	0.042	0.108	[−0.169, 0.253]	−0.201	0.105	[−0.408, 0.005]
Gender	−0.096	0.197	[−0.483, 0.290]	−0.807***	0.193	[−1.185, −0.428]
Grade	−0.166	0.161	[−0.481, 0.149]	0.188	0.157	[−0.121, 0.496]
Educational level	−0.128	0.227	[−0.573, 0.317]	0.696**	0.222	[0.261, 1.132]
Physical activity	0.027***	0.005	[0.018, 0.036]	−0.008	0.004	[−0.017, 0.001]
Positive mental health				−0.250***	0.018	[−0.284, −0.216]
*R* ^2^	0.014			0.070		
*F*	8.828***			39.097***		

**p < 0.01; ***p < 0.001. SE, Standard Error.

### Sensitivity analysis

To assess the robustness of our correlational findings against violations of normality, we conducted a bootstrap analysis with 5,000 resamples. The 95% percentile confidence intervals (CIs) confirmed the stability of all significant relationships. Critically, the positive correlation between physical activity and positive mental health (*ρ* = 0.143, 95% CI [0.108, 0.179]), the negative correlation between exercise and depression (*ρ* = −0.089, 95% CI [−0.125, −0.053]), and the strong negative correlation between positive mental health and depression (*ρ* = −0.362, 95% CI [−0.395, −0.328]) all remained significant, as their CIs excluded zero.

For the mediation analysis, we employed bias-corrected and accelerated (BCa) bootstrap intervals. The indirect effect of physical activity on depressive symptoms through positive mental health remained significant, and this indirect effect is small in magnitude: *ab* = −0.007, 95% BCa CI [−0.010, −0.004]. In completely standardized units, this corresponds to β_indirect_ = −0.028, 95% BCa CI [−0.039, −0.017]. Bootstrap intervals for all key paths (the *a* path from physical activity to positive mental health and the *b* path from positive mental health to depressive symptoms) were consistent with the conventional results presented in Table 4. Collectively, these analyses confirm that both the correlational and mediation findings are robust to the non-normal distribution of the data.

## Discussion

This study investigated the relationship between physical activity and depressive symptoms among Chinese college students. The results suggest a small indirect association consistent with a mediation pattern, where positive mental health may function as a mediator between physical activity and depressive symptoms. The following discussion will first interpret these findings in light of existing literature, then explore their theoretical and practical implications, and finally acknowledge the study’s limitations while suggesting directions for future research.

This study observed a significant inverse association between physical activity and depressive symptoms among university students, thereby providing support for hypothesis 1. These findings converge with established evidence on the activity-depression nexus (Ansari et al., 2025; Kandola et al., 2019; Schuch et al., 2018). This inverse association may manifest through experiential mechanisms where physical activity disrupts depressive cognition cycles. Physical activity attenuates rumination, thereby contributing to reduced depressive affect (Xu et al., 2025). An alternative explanatory pathway posits that physical activity fosters self-efficacy development, substantially mitigating depressive symptom trajectories through enhanced coping capacity (Chair et al., 2020). Emerging research indicates physical activity cultivates psychological capital—a multifaceted psychological construct that demonstrates significant alleviation of depressive symptoms (Luo et al., 2025). In the relationship between physical activity dosage and depressive symptoms, a study involving over 17,000 samples indicates that moderate-intensity physical activity is associated with a reduction in depressive symptoms (Ansari et al., 2025). Lower-intensity physical activity still enhance psychological well-being, while even single 5–10 min weekly sessions may yield cognitive-emotional improvements (Herbert, 2022).

Furthermore, this study suggests a small indirect association consistent with a mediation pattern between physical activity and depressive symptoms via positive mental health, and this indirect effect is small in magnitude, and hypothesis 2 was verified. Regular physical activity enhances positive mental health through psychosocial mechanisms. Studies have shown that aerobic exercise can enhance emotional regulation and stress resilience (Heijnen et al., 2016; Szuhany et al., 2015), and the improvement of these abilities has a positive impact on positive mental health (Rodríguez-Rojo et al., 2025). Behaviorally, physical activity provides mastery experiences that cultivate self-efficacy and competence beliefs (Chair et al., 2020; Gu et al., 2020)—core components of psychological capital (Youssef-Morgan and Luthans, 2015). Group-based physical activities further generate social synchrony through coordinated movement, promoting interpersonal trust and belonging (Tarr et al., 2015). These mechanisms collectively build the hedonic (positive affect) and eudaimonic (functioning) dimensions of mental health as defined by Keyes’ (2007) dual-continua model. Enhanced positive mental health disrupts depressive pathology through two primary pathways: (i) Cognitive reappraisal: Elevated positive affect broadens attentional scope (Friedman and Förster, 2010; Gable and Harmon-Jones, 2011; Lacey et al., 2021), enabling flexible reinterpretation of negative stimuli and disrupting rumination cycles (Joormann and Stanton, 2016). (ii) Social fortification: Flourishing mental health individuals exhibit increased social approach behaviors through physical activity, creating supportive networks that buffer against stress-induced depression (Cruwys et al., 2013). The results of the mediation analysis indicated that physical activity was not directly associated with depressive symptoms. Instead, its association was primarily explained by a significant, yet modest, indirect pathway through the enhancement of positive mental health.

Our research findings contribute to a deeper understanding at the theoretical level. They reveal a weak indirect association consistent with the existence of a mediating relationship between physical activity and depressive symptoms among college students. Positive mental health plays a modest mediating role in this process, although the indirect effect size is relatively small. This evidence suggests a complementary explanatory focus that incorporates a resource-building perspective alongside traditional deficit-reduction models. We outline a perspective wherein physical activity may contribute to emotional well-being in part by fostering positive psychological resources, such as stress resilience and self-efficacy (Chair et al., 2020; Heijnen et al., 2016; Szuhany et al., 2015). In this context, positive mental health can be considered a plausible explanatory construct, offering a pathway to integrate insights from positive psychology into the physical activity domain and thereby enriching the understanding of mental health promotion. This perspective points to potential implications. For instance, considering individuals with diminished positive mental health could inform the development of more nuanced preventive strategies. Our study thus highlights the rationale for exploring more tailored approaches in mental health promotion, where physical activity might be particularly considered for subgroups who could benefit most. Consequently, our primary theoretical contribution lies in proposing positive mental health as a useful conceptual focus for future research. Although the effect is statistically significant, the indirect effect size (*ab* = −0.007, β_indirect_ = −0.028) is small and likely of limited clinical relevance at the individual level; any population-level claims are speculative and require dedicated intervention and longitudinal studies in the future.

Although the data were collected from Chinese college students, the findings hold broader practical implications given two pressing public health concerns in this population. First, our survey indicates that 69.4% of Chinese college students report low physical activity levels. Second, 19.2% self-report depressive symptoms—a prevalence more than double the rate documented a decade ago (Zhang et al., 2013) and comparable to recent estimates in Chinese adolescent and collegiate populations (Wang et al., 2024a,b). Within the global college student population, the prevalence of depressive symptoms among Chinese students eclipsed rates in Lebanon (10.4%; El Khoury-Malhame et al., 2024), Croatia (9.8%; Milić et al., 2024), and Germany (17.3%; Erschens et al., 2024), yet trailed levels reported in South Africa (67.8%; Vagiri et al., 2025), Ukraine (47.0%; Pinchuk et al., 2024), Canada (38.9%; Vereschagin et al., 2024), Saudi Arabia (33.6%; Alsalman et al., 2024), and South Sudan (33.2%; Almadani et al., 2024). Despite not representing the most severe global prevalence, the marked decade-long escalation in depressive symptoms among Chinese college students necessitates urgent prioritization by college health authorities. Meanwhile, our study provides a mechanistic link between physical activity and depressive symptoms by identifying positive mental health as a significant mediator. While the unstandardized indirect effect (*ab* = −0.007) may appear modest at the individual level, its public health implications warrant consideration. Applied to a population scale, even small reductions in depressive symptoms can translate to meaningful benefits. For instance, assuming a linear relationship, if a campus-wide intervention increased physical activity levels across the student population, the mediated effect through positive mental health could contribute to a measurable decrease in the prevalence of clinically significant depressive symptoms. This aligns with the prevention paradox perspective, where small effects at the individual level yield substantial population-level impact (Rose, 1985).

Therefore, we propose a multi-tiered strategy for colleges to integrate physical activity into mental health promotion: Firstly, universal, environmental-level strategies: Colleges should develop conducive spaces, facilities, and engaging sports programs to boost overall participation. Implementing structured, school-wide physical activity plans can help shift the population norm toward moderate-intensity activity. Secondly, selective, knowledge-based strategies: Given that limited mental health literacy is a risk factor for depression (Huang et al., 2021; Lam, 2014), colleges should provide effective mental health education courses to enrich students’ knowledge and help them recognize the value of activities for well-being. Thirdly, indicated, individualized strategies: For students needing support, mental health professionals should consider integrating physical activity prescriptions into personalized care plans. These interventions should highlight physical activity’s dual role in enhancing positive psychological states while disrupting depressive cycles. This comprehensive approach harnesses physical activity’s potential to build resilience, foster holistic development, and create sustainable coping resources for academic and personal success.

## Limitations and future research

Several limitations of the current study should be acknowledged, along with corresponding future research directions. First, the cross-sectional design introduces temporal ambiguity in the mediation pathways, as it prevents confirmation of causal sequences. To address this, future longitudinal studies using experience sampling methods could track weekly fluctuations in activity, mental health states, and mood to establish the temporal precedence of mediation. Second, there is contextual specificity in mental health metrics: self-reported positive mental health measures may not fully capture culturally distinct manifestations of well-being in Chinese students (e.g., collectivist resilience). Mixed-methods research combining physiological markers (e.g., cortisol levels) with in-depth interviews could help identify contextually relevant well-being indicators. Third, the internal consistency of the PARS-3 scale was modest (α = 0.634), potentially affecting measurement precision. Future studies should seek to validate or adapt the PARS-3 scale to improve its reliability in the target population. Fourth, the use of a convenience sampling frame limits the generalizability of the results; to address this, future work should seek to replicate these findings in more diverse, probabilistically sampled populations. Fifth, the variance in positive mental health explained by physical activity and the covariates in our model was low (*R*^2^ = 0.014). This indicates that while physical activity is a statistically significant predictor of positive mental health, numerous other unmeasured factors (e.g., personality traits, social relationships) likely contribute substantially to an individual’s level of positive mental health (Piñar-Rodríguez et al., 2024; Su et al., 2020). It is important to note that in mediation analysis, the primary statistical test concerns the significance of the indirect effect (*a × b*), not the overall variance explained in the mediator. A significant *a* path (as found here) is sufficient to establish the first leg of the mediation, even within a model with a modest *R*^2^ for the mediator (Hayes, 2018; Preacher and Hayes, 2008). Future research should incorporate a broader nomological net of predictors to build more comprehensive models of positive mental health. Furthermore, as the sample consisted exclusively of Chinese university students, the cultural specificity of the findings must be considered. The applicability of the proposed mediation model to other populations requires verification. Future research should therefore prioritize cross-cultural validation. This involves two key steps: first, establishing the cross-cultural measurement invariance of the core constructs; second, conducting comparative studies to test whether the strength of the mediation pathway varies across cultures and investigating the contextual factors that explain such variation.

## Conclusion

This study provides evidence consistent with a mediating role of positive mental health between physical activity and depressive symptoms among college students. While the mediated effect is modest in magnitude, this finding holds both theoretical and preventive significance. Theoretically, it shifts the explanatory focus from deficit reduction to resource building, identifying a specific psychological pathway. In practice, it supports the implementation of population-wide physical activity promotion as a preventive strategy, where even small individual effects can yield meaningful public health benefits. Future experimental and longitudinal research is needed to establish causality and explore additional underlying mechanisms. Due to the limitations of this study, the research results should be interpreted with caution.

## Data Availability

The raw data supporting the conclusions of this article will be made available by the authors, without undue reservation.

## Footnotes

1
http://www.wjx.cn
